# Macrophage Activation Syndrome Associated with Adult-Onset Still's Disease Successfully Treated with Anakinra

**DOI:** 10.1155/2016/3717392

**Published:** 2016-10-12

**Authors:** Aswini Kumar, Hiroshi Kato

**Affiliations:** SUNY Upstate Medical University, 750 E. Adams Street, Syracuse, NY 13202, USA

## Abstract

Macrophage activation syndrome (MAS) is a potentially fatal complication of Adult-Onset Still's disease (Still's disease). Whereas an increasing body of evidence supports interleukin-1 (IL-1) blockade as a promising treatment for Still's disease, whether it is therapeutic for MAS associated with Still's disease remains unclear. We report a 34-year-old Caucasian man with one-decade history of TNF-blockade-responsive seronegative arthritis who presented with abrupt onset of fever, serositis, bicytopenia, splenomegaly, hepatitis, and disseminated intravascular coagulation. Striking hyperferritinemia was noted without evidence of infection, malignancy, or hemophagocytosis on bone marrow biopsy. NK cells were undetectable in the peripheral blood, whereas soluble IL-2 receptor was elevated. His multiorgan disease resolved in association with methylprednisolone pulse therapy, Anakinra, and a tapering course of prednisone. This case reinforces the notion that Still's disease is inherently poised to manifest MAS as one of the clinical phenotypes by shedding light on the role of IL-1 underlying both Still's disease and related MAS.

## 1. Introduction

Hemophagocytic lymphohistiocytosis (HLH) encompasses a spectrum of disease processes characterized by the accumulation of well-differentiated mononuclear cells with a macrophage phenotype exhibiting hemophagocytic activity. It clinically presents with a syndrome of excessive immune activation which often culminates in life-threatening multiorgan disease characterized by fever, pancytopenia, splenomegaly, hepatitis, encephalopathy, and coagulopathy. HLH is classified into primary (familial) or secondary HLH, depending on the presence or absence of homozygous deficiency of cytolytic pathway proteins [1–3]. Specifically, HLH which occurs in a patient with systemic onset juvenile idiopathic arthritis (sJIA), Still's disease, or other rheumatic diseases is termed macrophage activation syndrome (MAS). However, flare of these rheumatic diseases and MAS are largely indistinguishable based on the clinical and laboratory grounds, posing a significant diagnostic challenge to clinicians. In this regard, in addition to the 10% risk of developing overt MAS as part of sJIA, another 30–40% of sJIA patients may have subclinical MAS during disease flare that can eventually culminate in overt MAS [4, 5]. These observations may allow one to speculate that sJIA and Still's disease are inherently programmed to manifest MAS in a substantial portion of cases. Nonetheless, it remains unclear whether therapeutic approach to Still's disease is applicable to MAS secondary to Still's disease or HLH-specific treatment, such as HLH-94 or HLH-2004 protocol [6], should be employed depending on the severity of disease. As an example, while IL-1 blockade has been attracting much attention as a promising therapy for Still's disease, it is not known whether it is also an effective treatment for MAS associated with Still's disease as only a few such cases have been reported to date. We herein report a 34-year-old man who developed MAS as part of Still's disease which was successfully treated with Anakinra without HLH-specific protocol, solidifying the aforementioned concept that MAS may be one of the inherently programmed clinical phenotypes of Still's disease.

## 2. Case Presentation

34-year-old Caucasian man was admitted to our hospital for two-week history of multisystem illness characterized by fever, nonexertional chest pain, abdominal pain, severe liver disease, thrombocytopenia, and coagulopathy.

The patient had been in his usual state of health until two weeks prior to the admission, when he hit a deer upon motorcycle accident and suffered sinus and rib fractures. Following the accident, he developed worsening fever, night sweats, malaise, diffuse myalgia, nonexertional chest pain, and abdominal pain.

He has suffered one-decade history of seronegative inflammatory arthritis affecting the small and large joints in a symmetrical distribution. His arthritis had been in remission in association with etanercept 50 mg SQ weekly prescribed by an outside rheumatologist, which was discontinued three years prior to this presentation as he had developed blurry vision, gait disturbance, and paresthesia in the distal lower extremities. These symptoms resolved upon cessation of etanercept and thus were attributed to etanercept. Since then, he has been treated with hydroxychloroquine 200 mg PO daily. While the patient did not recall major flare of arthritis until the current presentation, he had suffered persistent mild to moderate arthralgia. His arthritic symptoms have often been accompanied by fever, sore throat, pleurisy, and evanescent erythematous rash. His maternal grandfather and aunt had rheumatoid arthritis. His paternal grandmother had myasthenia gravis. Besides the contact with a deer upon the accident, he denied exposure to animals or sick contact.

On examination, the patient was in moderate distress. The temperature was 39.8°C, the blood pressure was 130/79 mm Hg, the pulse was 94 beats per minute, and the oxygen saturation was 98% while he was breathing ambient air. Conjunctivae were pale and icteric. Superficial lymph nodes were not palpable. Breath sounds were diminished in bilateral lower 1/3 of the lungs. There were no pericardial friction rubs. Abdomen was diffusely tender and spleen tip was palpable. Blanching erythematous macular rash was noted over the right shoulder. There was no synovitis. The remainder of the examination was unremarkable.

Laboratory studies showed leukocyte count at 14,900/*μ*L (reference range: 4000–10,000/*μ*L; reference range is provided in the parentheses in the following laboratory studies), neutrophils 12,900/*μ*L (1800–7000/*μ*L), hemoglobin 11.4 g/dL (13.5–18 g/dL), platelets 21,000/*μ*L (150,000–400,000/*μ*L), and an erythrocyte sedimentation rate (ESR) at 103 mm/h (0–15 mm/h). His hemoglobin subsequently dropped and reached its nadir of 8.3 g/dL on the 3rd hospital day. Chemistries showed serum creatinine at 0.7 mg/dL (0.5–1.2 mg/dL), lactate dehydrogenase 441 U/L (112–225 U/L), aspartate aminotransferase 306 U/L (<38 U/L), alanine aminotransferase 343 U/L (<41 U/L), alkaline phosphatase 117 U/L (40–129 U/L), albumin 2.9 g/dL (3.4–4.8 g/dL), total bilirubin 3.8 mg/dL (0.1–1.0 mg/dL), and direct bilirubin 2.9 mg/dL (0–0.3 mg/dL). Ferritin was markedly elevated at 4542 ng/mL (30–400 ng/mL). Triglyceride was 125 mg/dL (<200 mg/dL). Coagulation studies showed PT-INR at 1.61, PT 20.0 seconds (12.5–14.9 seconds), aPTT 78.5 seconds (24.6–33.4 seconds), D-dimer 13.02 *μ*g/mL (<0.50 *μ*g/mL), and fibrinogen 534 mg/dL (190–450 mg/dL), which yielded a disseminated intravascular coagulation (DIC) score at 6, consistent with overt DIC [7]. Urinalysis did not indicate infection. CT showed bilateral pleural effusion, mild splenomegaly, and a small amount of ascites. The patient was started on piperacillin/tazobactam 3.375 grams IV every 8 hours and vancomycin 1 gram IV every 12 hours as well as intravenous hydrocortisone 100 mg every 8 hours. Given the thrombocytopenia and severe liver disease in the setting of potential tick exposure, empirical treatment with doxycycline 100 mg IV every 12 hours was pursued. Two sets of negative blood cultures and unremarkable transthoracic echocardiogram ruled out endocarditis. Urine histoplasma antigen was not detected. Peripheral smear did not show intracytoplasmic morulae. Serologies for* E. chaffeensis* and* R. rickettsii* were negative as were those for hepatitis B, hepatitis C, and HIV. Bone marrow biopsy did not reveal evidence of hemophagocytosis or hematological malignancy. ANA was positive at 1/1250 dilution (speckled pattern) while double-stranded DNA, Smith, Ro, La, and RNP antibodies were negative. C3 and C4 were 73 mg/dL (90–180 mg/dL) and 7 mg/dL (10–40 mg/dL), respectively. Lupus anticoagulant was detected based on hexagonal phase phospholipid dilution assay and dilute russell viper venom time, as were Cardiolipin IgG 30 GPL (0–14 GPL), Cardiolipin IgM 128 MPL (0–12 MPL), Cardiolipin IgA 133 APL (0–11 APL), Beta-2 Glycoprotein IgM > 150 SMU (<20 SMU), and Beta-2 Glycoprotein IgA 54 SAU (<20 SAU). Nonetheless, there was no evidence of thromboembolic disease on CT of chest/abdomen/pelvis, and peripheral blood smear did not show microangiopathic picture. Antineutrophil cytoplasmic antibody was negative as were rheumatoid factor, cyclic citrullinated antibody, and cryoglobulin. The patient was collectively diagnosed with Still's disease presenting with serositis, hepatitis, and DIC and was started on intravenous methylprednisolone 1 gram daily for 3 days as well as Anakinra 100 mg SQ daily. His overall condition significantly improved and he was discharged on the 9th hospital day on Anakinra and a tapering course of prednisone. After his discharge, natural killer (NK) cells were reported to be undetectable in his peripheral blood. Blood specimen for soluble IL-2 receptor was sent on the 5th hospital day, which was mildly elevated at 1598 units/mL (45–1105 units/mL). Accordingly, he met 5 out of 8 criteria for hemophagocytic lymphohistiocytosis (HLH); fever, splenomegaly, bicytopenia, hyperferritinemia, and no NK cell [6]. Regardless, the bicytopenia, liver disease, and coagulopathy resolved in two months without HLH-specific therapy. The increased ferritin and ESR significantly improved in 6 weeks at 192 ng/mL and 24 mm/h, respectively. During the course of steroid tapering, the patient developed a flare of polyarthritis. As such, methotrexate was added and allowed him to taper off prednisone. However, he subsequently self-discontinued Anakinra and started to develop recurrent flare of arthritis. As he did not wish to resume self-injection of Anakinra, methotrexate was switched to mycophenolate mofetil. Since then, the patient has remained well without major flare of arthritis.

## 3. Discussion

When we first evaluated this patient, sepsis is our leading concern, and the patient received empirical antibiotics accordingly. Indeed, sepsis and MAS are often indistinguishable based on the clinical grounds. Additionally, the fever, thrombocytopenia, and hepatitis raised a concern for tick-borne illness, in particular ehrlichiosis, given his potential exposure, and as such, he received empirical doxycycline. Nonetheless, extensive infectious disease work-up was nonrevealing including blood cultures, echocardiogram, peripheral blood smear, and serologies for* E. chaffeensis* and* R. rickettsia*. Furthermore, the patient's overall clinical conditions improved in association with immunosuppression, rendering infectious etiology unlikely.

Once infectious disease was ruled out, a flare of Still's disease was our leading differential diagnosis in view of the preexisting illness characterized by fever, sore throat, seronegative arthritis, and evanescent rash as well as the leukocytosis, serositis, hepatitis, and coagulopathy on the current presentation. He was later found to have undetectable NK cells and elevated soluble IL-2 receptor and thus fulfilled the 5 out of 8 criteria for HLH: fever, splenomegaly, bicytopenia, hyperferritinemia, and no NK cell [6]. The leukocytosis likely reflected a concurrent flare of Still's disease and rendered SLE less likely despite the serological findings as discussed below. Our patient did not have hepatomegaly or lymphadenopathy, which are relatively common, but not necessarily present in MAS [8, 9], and the HLH-2004 criteria do not rely on these features [6]. Likewise, hemophagocytosis was not observed in the bone marrow; however, it is important to keep in mind that hemophagocytic picture was noted only in 70% of patients in a recent retrospective case series [10]. In fact, the diagnosis of HLH does not necessarily require the presence of hemophagocytosis in the bone marrow [6]. A major challenge in the diagnosis of MAS in adult patients with rheumatic disease is the lack of definitive classification criteria in this specific population. In this regard, whether the HLH-2004 criteria can be extrapolated to MAS remains to be investigated. Our case fulfilled the latest classification criteria for MAS proposed by Ravelli et al. and Kostik et al. [8, 9]; however, it is important to keep in mind that these criteria were developed for pediatric population, that is, sJIA, but not for Adult-Onset Still's disease [8].

There were serological features reminiscent of SLE, including ANA, APLA, and hypocomplementemia. However, the constellation of clinical features prior to the current presentation, including fever, sore throat, and evanescent erythematous rash, as well as the neutrophilic leukocytosis would rather point to Still's disease than SLE [11–13]. With regard to the serological findings, it is important to keep in mind that TNF-blocking agents induce a wide variety of autoantibodies including ANA and APLA [14, 15]. Additionally, an anecdotal case series reported a high incidence of APLA in Still's disease [16]. Given the severity of multiorgan illness, catastrophic antiphospholipid syndrome was an important differential diagnosis. However, there was no evidence of thromboembolic disease on the CT-chest/abdomen/pelvis, and his peripheral blood smear did not show microangiopathic picture. While he was found to have hypocomplementemia, there was no evidence of immune-complex-driven pathology such as glomerulonephritis, which together with the severity of hepatitis led us to reason that the hypocomplementemia likely reflected reduced complement production in the setting of liver disease.

His arthritis had been in remission on etanercept and previous open label trials suggest the efficacy of TNF-blocking therapy in Still's disease [17, 18]. Additionally, his multiorgan disease resolved on steroid and Anakinra without HLH-specific treatment. We collectively conclude that full-blown manifestations of Still's disease had been partially masked by prior TNF-blocking therapy and that cessation of TNF-blocking agent and the catastrophic event, that is, motorcycle accident, likely unveiled a flare of Still's disease culminating in MAS.

The original paradigm of primary versus secondary HLH has recently been challenged as an increasing body of evidence points to the overlapping genetic background between MAS and familial HLH [19, 20]. Nonetheless, it is well accepted that there are certain medical conditions which predispose a patient to HLH, including infection, lymphoid malignancy, and rheumatic disease, in particular sJIA and Still's disease. It is plausible that trigger-specific or underlying disease-specific therapy is the most essential component of treatment and obviates the need for HLH-directed protocol, such as HLH-94 or HLH-2004, in the majority of cases in this category of HLH. However, such a hypothesis has not been rigorously examined to date, rendering optimal therapeutic approach to MAS obscure. Regarding the MAS in the setting of Still's disease, successful treatment with intravenous immunoglobulin, methotrexate, cyclosporine, or cyclophosphamide with/without steroid has been reported in anecdotal case series [21, 22]. However, most of these drugs are globally immunosuppressive and their off-target effects lead to substantial morbidity and mortality in a large portion of patients. Therefore, it has been an unmet need to precisely define disease mechanisms and develop minimally toxic target-specific therapeutics in the care of Still's disease and related MAS. Serum from sJIA patients induced the transcription of IL-1 in peripheral blood mononuclear cells [23]. A growing body of evidence sheds light on the prominent role of IL-1 as a driver of sJIA as well as Still's disease [24, 25]. Further, successful application of IL-1-signaling blockade, such as Anakinra, has been increasingly reported in those with MAS secondary to sJIA [26]. In HLH, genes associated with IL-1-signaling pathway are upregulated [27]. Nonetheless, the precise role of IL-1 in the pathogenesis of MAS has remained unclear [28]. sJIA and Still's disease share many clinical, laboratory, and immunopathological features and are distinguished solely based on the age of onset. However, to the best of our knowledge, successful treatment with IL-1 blockade has been reported only in three cases of MAS in the setting of Adult-Onset Still's disease [29–31]. Our patient received Anakinra concurrently with high dose steroid. As such, one may argue that his clinical improvement could have been attributed to the combination of the two drugs or steroid. However, we would like to point out that he suffered recurrent flare of arthritis once he had self-discontinued Anakinra. Thus, we reason that IL-1 was likely playing an important role in his disease. Finally, it is plausible that certain patients with MAS might benefit from HLH-directed treatment as opposed to IL-1 blockade even in the setting of Still's disease. To precisely identify such patients, we must await future studies which elucidate biomarkers indicative of upregulated IL-1-signaling cascade in this population as well as correlation of such biomarkers to clinical response to IL-1 blockade.

In summary, our case not only reemphasizes the critical role of IL-1 in the pathogenesis of Still's disease, but also adds further evidence to the notion that MAS is an inherent attribute of Still's disease by demonstrating that both diseases likely share IL-1-driven inflammatory pathway as a common disease mechanism.

## References

[1] Stepp S. E., Dufourcq-Lagelouse R., Le Deist F. (1999). Perforin gene defects in familial hemophagocytic lymphohistiocytosis. *Science*.

[2] Feldmann J., Callebaut I., Raposo G. (2003). Munc13-4 is essential for cytolytic granules fusion and is mutated in a form of familial hemophagocytic lymphohistiocytosis (FHL3). *Cell*.

[3] Zhang K., Chandrakasan S., Chapman H. (2014). Synergistic defects of different molecules in the cytotoxic pathway lead to clinical familial hemophagocytic lymphohistiocytosis. *Blood*.

[4] Behrens E. M., Beukelman T., Paessler M., Cron R. Q. (2007). Occult macrophage activation syndrome in patients with systemic juvenile idiopathic arthritis. *Journal of Rheumatology*.

[5] Bleesing J., Prada A., Siegel D. M. (2007). The diagnostic significance of soluble CD163 and soluble interleukin-2 receptor *α*-chain in macrophage activation syndrome and untreated new-onset systemic juvenile idiopathic arthritis. *Arthritis and Rheumatism*.

[6] Henter J.-I., Horne A., Aricó M. (2007). HLH-2004: diagnostic and therapeutic guidelines for hemophagocytic lymphohistiocytosis. *Pediatric Blood and Cancer*.

[7] Taylor F. B., Toh C. H., Hoots W. K., Wada H., Levi M. (2001). Towards definition, clinical and laboratory criteria, and a scoring system for disseminated intravascular coagulation. *Journal of Thrombosis and Haemostasis*.

[8] Ravelli A., Minoia F., Davi S. (2016). Classification criteria for macrophage activation syndrome complicating systemic juvenile idiopathic arthritis: a European league against rheumatism/American College of Rheumatology/Paediatric Rheumatology International Trials Organisation Collaborative Initiative. *Annals of the Rheumatic Diseases*.

[9] Kostik M. M., Dubko M. F., Masalova V. V. (2015). Identification of the best cutoff points and clinical signs specific for early recognition of macrophage activation syndrome in active systemic juvenile idiopathic arthritis. *Seminars in Arthritis and Rheumatism*.

[10] Rivière S., Galicier L., Coppo P. (2014). Reactive hemophagocytic syndrome in adults: a retrospective analysis of 162 patients. *The American Journal of Medicine*.

[11] Petri M., Orbai A.-M., Alarcõn G. S. (2012). Derivation and validation of the systemic lupus international collaborating clinics classification criteria for systemic lupus erythematosus. *Arthritis & Rheumatism*.

[12] Hochberg M. C. (1997). Updating the American College of Rheumatology revised criteria for the classification of systemic lupus erythematosus. *Arthritis & Rheumatism*.

[13] Yamaguchi M., Ohta A., Tsunematsu T. (1992). Preliminary criteria for classification of adult Still's disease. *Journal of Rheumatology*.

[14] Atzeni F., Turiel M., Capsoni F., Doria A., Meroni P., Sarzi-Puttini P. (2005). Autoimmunity and anti-TNF-*α* agents. *Annals of the New York Academy of Sciences*.

[15] Jonsdottir T., Forslid J., van Vollenhoven A. (2004). Treatment with tumour necrosis factor *α* antagonists in patients with rheumatoid arthritis induces anticardiolipin antibodies. *Annals of the Rheumatic Diseases*.

[16] Milchert M., Fisher K., Fliciński J., Ostanek L., Brzosko M. (2010). High prevalence of anti-beta-2 glycoprotein-I and anti-prothrombin antibodies in adult-onset Still's disease. Comment on ‘portal vein thrombosis in adult-onset Still's disease: a case report and literature review’. *Rheumatology International*.

[17] Kraetsch H. G., Antoni C., Kalden J. R., Manger B. (2001). Successful treatment of a small cohort of patients with adult onset of Still's disease with infliximab: first experiences. *Annals of the Rheumatic Diseases*.

[18] Husni M. E., Maier A. L., Mease P. J. (2002). Etanercept in the treatment of adult patients with Still's disease. *Arthritis & Rheumatism*.

[19] Zhang K., Biroschak J., Glass D. N. (2008). Macrophage activation syndrome in patients with systemic juvenile idiopathic arthritis is associated with MUNC13-4 polymorphisms. *Arthritis & Rheumatism*.

[20] Hazen M. M., Woodward A. L., Hofmann I. (2008). Mutations of the hemophagocytic lymphohistiocytosis-associated gene UNC13D in a patient with systemic juvenile idiopathic arthritis. *Arthritis and Rheumatism*.

[21] Hot A., Toh M.-L., Coppéré B. (2010). Reactive hemophagocytic syndrome in adult-onset still disease: clinical features and long-term outcome: a case-control study of 8 patients. *Medicine*.

[22] Arlet J.-B., Huong D. L. T., Marinho A. (2006). Reactive haemophagocytic syndrome in adult-onset Still's disease: a report of six patients and a review of the literature. *Annals of the Rheumatic Diseases*.

[23] Pascual V., Allantaz F., Arce E., Punaro M., Banchereau J. (2005). Role of interleukin-1 (IL-1) in the pathogenesis of systemic onset juvenile idiopathic arthritis and clinical response to IL-1 blockade. *Journal of Experimental Medicine*.

[24] Nordström D., Knight A., Luukkainen R. (2012). Beneficial effect of interleukin 1 inhibition with anakinra in adult-onset Still's disease. An open, randomized, multicenter study. *Journal of Rheumatology*.

[25] Quartier P., Allantaz F., Cimaz R. (2011). A multicentre, randomised, double-blind, placebo-controlled trial with the interleukin-1 receptor antagonist anakinra in patients with systemic-onset juvenile idiopathic arthritis (ANAJIS trial). *Annals of the Rheumatic Diseases*.

[26] Miettunen P. M., Narendran A., Jayanthan A., Behrens E. M., Cron R. Q. (2011). Successful treatment of severe paediatric rheumatic disease-associated macrophage activation syndrome with interleukin-1 inhibition following conventional immunosuppressive therapy: case series with 12 patients. *Rheumatology*.

[27] Sumegi J., Barnes M. G., Nestheide S. V. (2011). Gene expression profiling of peripheral blood mononuclear cells from children with active hemophagocytic lymphohistiocytosis. *Blood*.

[28] Schulert G. S., Grom A. A. (2014). Macrophage activation syndrome and cytokine-directed therapies. *Best Practice & Research: Clinical Rheumatology*.

[29] Loh N. K., Lucas M., Fernandez S., Prentice D. (2012). Successful treatment of macrophage activation syndrome complicating adult Still disease with anakinra. *Internal Medicine Journal*.

[30] Youssef J., Lazaro E., Blanco P., Viallard J.-F. (2008). Blockade of interleukin 1 receptor in Still's disease affects activation of peripheral T-lymphocytes. *The Journal of Rheumatology*.

[31] Durand M., Troyanov Y., Laflamme P., Gregoire G. (2010). Macrophage activation syndrome treated with anakinra. *The Journal of Rheumatology*.

